# Supplementary material for: Difficulty in chirality recognition for Transformer architectures learning chemical structures from string representations
Source: Nat Commun. 2024 Feb 16;15:1197. doi: 10.1038/s41467-024-45102-8 (PMC10873378; doi:10.1038/s41467-024-45102-8)
Supplement: Supplementary file 1 — Supplementary Information [file 41467_2024_45102_MOESM1_ESM.pdf]

Supplementary information for “Difficulty in chirality recognition for Transformer architectures learning chemical structures from string representations”

Yasuhiro Yoshikai[1,#] **Tadahaya Mizuno**[1,*,#]
Shumpei Nemoto[1] **Hiroyuki Kusuhara**[1]

[1] Laboratory of Molecular Pharmacokinetics, Graduate School of Pharmaceutical Sciences, The University of Tokyo, 7-3-1 Hongo, Bunkyo, Tokyo, Japan
[#] These authors contributed equally.
[*] **Corresponding Author:** Tadahaya Mizuno - tadahaya@gmail.com

Supplementary Notes

1 Related works

Chemical language models can be roughly classified into 3 categories based on their applications: structure generation (e.g., de novo drug design and retrosynthesis), end-to-end property prediction, and descriptor generation [1–15]. The difference between the former 2 and descriptor generation from a machine learning perspective is the need for prior information. The former ones are supervised methods, whereas descriptor generation is unsupervised in general. In this regard, chemical language models for descriptor generation, which is the main topic of this study, are models that purely learn chemical structures.

Research on chemical language models with neural networks was first started in the study of Gómez-Bombarelli et al. [16],which applied a variational autoencoder (VAE)[17]structure to SMILES strings. They tried to represent the distribution of SMILES strings of various molecules by VAE structure, using GRU as the encoder and the decoder. Winter et al. [18] generated molecular descriptors by GRU model trained for translation tasks between 2 different SMILES representations of molecules.

While the studies by Gómez-Bombarelli et al. or Winter et al. adopted the RNN model for chemical language models, the most prosperous model in the NLP study is recently becoming the Transformer [19]. The key feature of the Transformer model is its attention structure, in which the model processes each word attending to all words in the sentence (self-attention) or other information (cross-attention) globally. Various derivative methods have been devised based on this structure such as bidirectional encoder representations from transformers (BERT) [20–23]. Honda et al. [24] trained a Transformer by making it translate 2 different types of SMILES and conducted property prediction by descriptor pooled from intermediate memory in the model. Fabian et al. [25], based on BERT, pretrained the model not only with NLP tasks adopted in the original BERT model but also with domain-specific tasks such as fixed molecular descriptor prediction, and reported that these additional training tasks improved the performance of the model on downstream cheminformatics tasks. Irwin et al. [15] pretrained a Transformer by predicting masked SMILES, translating different types of SMILES, and fine-tuned the model to predict molecular properties. The Transformer architecture is widely utilized as chemical language models for other objectives such as molecular generation [1–5] and retrosynthesis prediction [6–9].

There are several studies to modify the Transformer structure to be suitable for recognizing chemical structures by chemical language models. One potent strategy is to combine a Transformer, or its key feature, self-attention mechanism, with 2D graph-based architecture because 2D graph representation directly provides chemical structures and high visibility of them[10–12,14,26–28]. However, these studies focused on recognizing chemical structures in end-to-end tasks such as molecular property prediction,

depending on specific tasks. To the best of our knowledge, no studies have focused on recognizing chemical structures by chemical language models for descriptor generation, which purely learns a wide variety of chemical structures.

As will be discussed later, this paper argues that the chirality of molecules has a substantial influence on the training of the Transformer. Stereochemistry, including chirality, has been sometimes ignored in previous studies on cheminformatics[18], especially when graph-based models are used, but some studies suggests that it is beneficial to incorporate information on stereoisomerism of compounds to some cheminformatics tasks. Lagnajit et al.[29] proposed new method for aggregating message passing neural network which takes chirality into account and showed improvement on protein-ligand docking prediction. Adams et al.[30] designed 3D GNN which is invariant to rotation but not to chirality, and achieved state of the art scores on chiral-related tasks.

2 Dimension-wise similarity of MACCS keys

In Partial/overall structure recognition of the Transformer in learning progress Section in the main paper, the similarities of ECFP and MACCS keys between predicted/targeted molecules were calculated, and early saturation of similarities was observed. MACCS keys are 166-bit fingerprints each of which represents whether the molecule has a certain predefined substructure. To investigate what kind of substructures are easy or difficult for the model to understand, we calculated the dimension-wise similarities between prediction and target. Here, we did not exclude molecules with invalid prediction, but instead calculated the ratio of molecules that were validly decoded and whose MACCS key bits matched between the predicted and target structures, relative to all molecules.

For each dimension *i*:

			Target Molecule	
			MACCS*i*= 0	MACCS*i*= 1
Prediction	Invalid		A	D
	Valid	MACCS*i*= 0	B	E
		MACCS*i*= 1	C	F

$$Ratio_i^0 = \frac{B}{A + B + C}$$

$$Ratio_i^1 = \frac{F}{D + E + F}$$

These scores can also remedy a limitation in the metric of the main paper which excluded molecules with invalid prediction and therefore may have filtered out complicated molecules and overestimated the similarity of fingerprints. Supplementary Figures 14 and 15 show the score for all dimensions in MACCS keys compared with their frequency (the ratio of molecules with bit 1 in each dimension). The result showed that no remarkable tendency or dimension was observed except that the ratio of correct 0/1 fingerprint is correlated to the frequency of 0/1 fingerprint in target molecules. As for temporal transition, the accuracy for most dimensions had converged to 1.0 by step 6,000, meaning almost complete reproduction of substructures. These results also support that partial structures are understood more rapidly than overall structures by the Transformer model.

3 Structure recognition and downstream task performance when stagnation occurred

In Stagnation of perfect accuracy in learning chemical structures Section in the main manuscript, we found that the perfect accuracy of the model is sometimes trapped, apparently depending on the initial weight of the model. In this section, we studied whether our findings in Sections Partial/overall structure recognition of the Transformer in learning progress and Downstream task performance in the learning progress are true for this stagnated model. We used the models in the training with initial weight #2 and iteration order #1, whose perfect accuracy transition is shown in Supplementary Figure 16a. We calculated the Tanimoto similarity of MACCS keys and ECFP and conducted molecular property prediction tasks for models when perfect accuracy reached 0.2, 0.5, 0.7, 0.9, 0.95, and 0.98 and models at steps 0, 4,000, and 80,000, just as we did in the main paper. Supplementary Figure 16b shows the Tanimoto similarity of MACCS keys and ECFP. It shows that the similarity of all fingerprints rose to nearly 1.0 at the early steps of training, while perfect accuracy and loss function is yet to converge. This result is consistent with that in Partial/overall structure recognition of the Transformer in learning progress Section. Regarding the performance of descriptors on downstream tasks, the performance did not rise in the training (Supplementary Figures 17 and 18), corresponding to the result in Downstream task performance in the learning progress Section. These results support what was suggested in the main paper, i.e., that the partial structure of the molecule

is rapidly understood by the model and the performance of the descriptor generated by the model did not change by training.

4 Training by balanced dataset about "@" and "@@" tokens

The training dataset was used in the main paper was slightly biased about chiral tokens; more "@@" tokens were contained than "@" tokens. In order to clarify whether this bias is the reason for stagnation, we prepared the training dataset with balanced number of "@" and "@@" tokens and trained the model with it. We first sampled molecules from ZINC-15 in a stratified way about the length of molecules (this time "@@" was treated as a single character not to be biased). Then some of the sampled and filtered molecules were downsampled so that the distribution of "@" and "@@" tokens in canonical SMILES is symmetrical for each length. The accumulated frequency of "@" and "@@" tokens in the dataset was shown in Supplementary Figure 19a. The model was trained with this model from 5 different initial weights. The result showed that stagnation did occur in some cases (Supplementary Figure 19b). indicating that the imbalance of "@" and "@@" tokens is not the cause of difficulty and stagnation in learning chirality.

5 Training of InChI-to-SMILES translation

The molecules extracted and preprocessed from ZINC in Training a Transformer Section were used here, and we trained the Transformer model to translate InChI expression of the molecules into canonical SMILES of them. The experiments were conducted for 5 times with different initial weights. As we changed batch size according to the length of strings so that each batch contains about 25,000 tokens in this paper, relatively long InChI expression reduced batch size and extended the length of 1 epoch to 184,652.5 steps. We therefore trained the model for up to 200,000 steps, although we aborted training when perfect accuracy reached 0.95.

6 Experiments with models trained by a randomly sampled SMILES string

In the main manuscript, we sampled SMILES data for training and validation in a stratified way concerning the lengths of SMILES strings, which was found to enhance the translation accuracy of the trained model in our previous studies, but this sampling measure is not commonly used. To clarify whether our finding in the main manuscript depends on this sampling method, we trained the model with randomly sampled training and validation data. We filtered randomly sampled molecules as in the main paper, and 30,000,752 (about 30M) molecules were left. We sampled about 3% (8,998) molecules as test set and trained the model with the remaining molecules. 136,626 batches (68,313 steps) were contained in one epoch. We trained the model for 4 times with different initial weights and the same iteration order, and conducted some experiments in the main paper with one of the modSuplels. Supplementary Figure 20a shows the perfect accuracy for 4 trials. The result shows that stagnation did occur in some cases with this unstratified data. We then trained the model with another seed for 80,000 steps, which did not result in stagnation (Supplementary Figure 20b) and conducted some of the experiments in those sections. We compared the Tanimoto similarity of molecular fingerprints between prediction and target with steps, perfect accuracy, and loss function (Supplementary Figure 20c). The similarity almost saturated at step 10,000, where perfect accuracy and loss function had not converged. To sum up, no results remarkably different from those with stratified data were obtained, and it was suggested that the results in the main paper do not depend on how to sample the training data.

Supplementary Figures

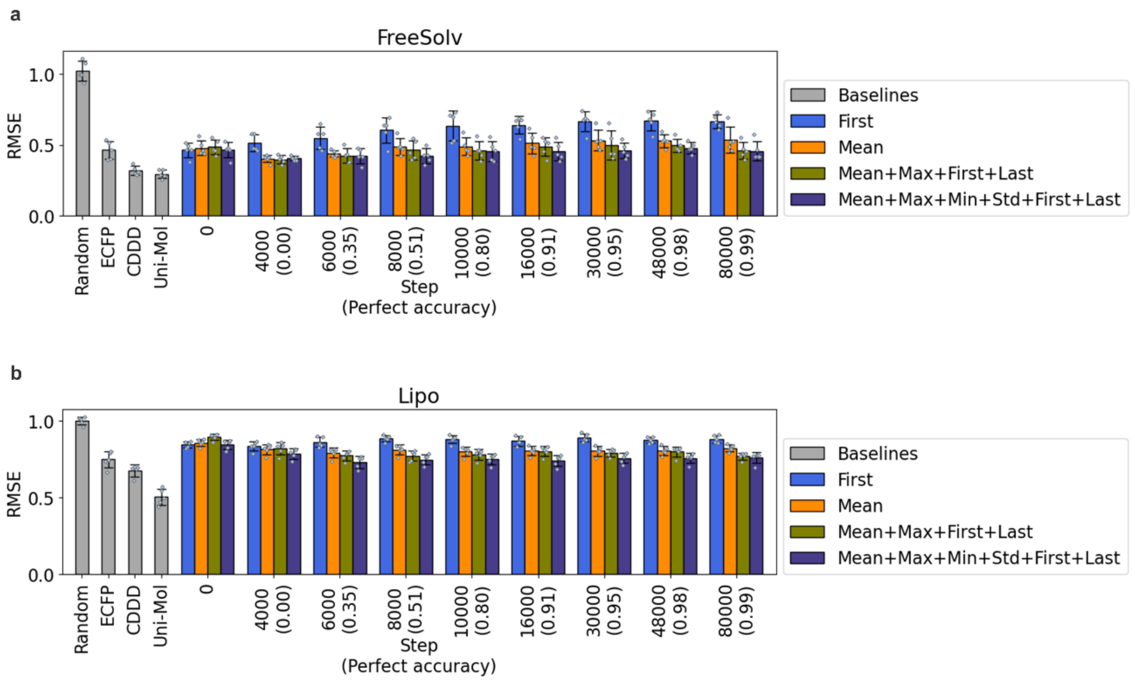

Supplementary Figure 1. Performance of descriptors on molecular property prediction (Regression task)

(a) RMSE score of prediction on FreeSolv dataset from descriptors of the model at different steps of training, for 4 different ways of pooling. Blue, mean; yellow, latent representation of the first token; red, concatenation of the indicated 4 aggregation methods; navy, concatenation of the indicated 6 aggregation methods. (b) RMSE score of prediction on Lipophilicity dataset from descriptors of the model at different steps of training for 4 different ways of pooling. Mean, unbiased standard deviation and data distribution of experiments for 5 folds split by recommended method in DeepChem[31] are shown as bar height, error bar length and gray dots, respectively. The metrics were determined based on MoleculeNet[32]. The perfect accuracy at each step is written down on the horizontal axis. Source data are provided as a Source Data file. RMSE, Root Mean Squared Error; FreeSolv, Free Solvation Database

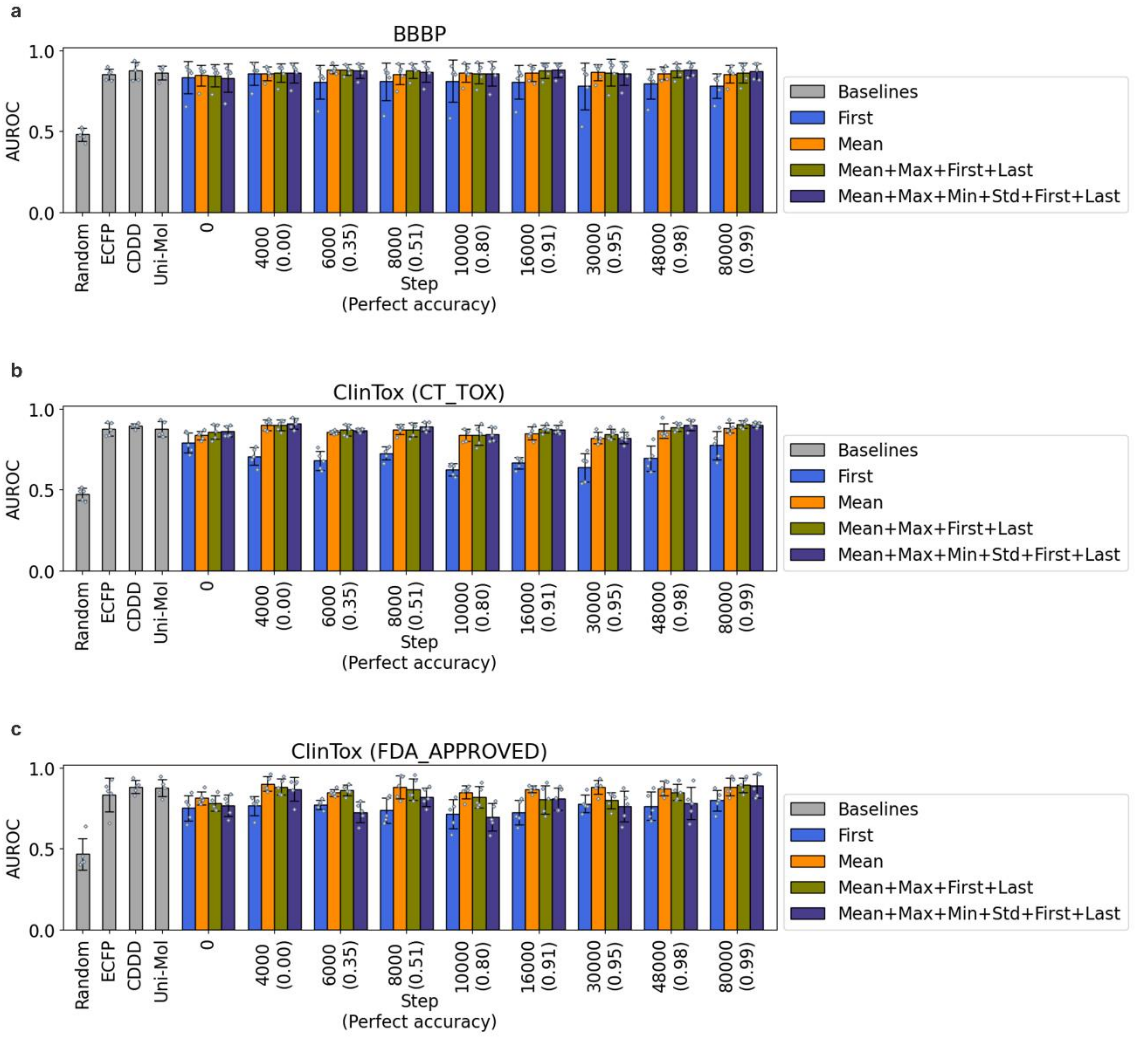

Supplementary Figure 2. Performance of descriptors on molecular property prediction (Classification task)

(a) AUROC score of prediction on BBBP dataset from descriptors of the model at different steps of training, for 4 different ways of pooling. Blue, mean; yellow, latent representation of the first token; red, concatenation of the indicated 4 aggregation methods; navy, concatenation of the indicated 6 aggregation methods. (b) AUROC score of prediction on ClinTox (failure of clinical trials for toxicity reasons) dataset from descriptors of the model at different steps of training for 4 different ways of pooling. (c) AUROC score of prediction on ClinTox (FDA approval) dataset from descriptors of the model at different steps of training for 4 different ways of pooling. Mean, unbiased standard deviation and data distribution of experiments for 5 folds split by recommended method in DeepChem[31] are shown as bar height, error bar length and gray dots, respectively. The metrics were determined based on MoleculeNet[32]. The perfect accuracy at each step is written down on the horizontal axis. Source data are provided as a Source Data file. AUROC, Area Under Receiver Operating Characteristic; BBBP, The blood-brain barrier penetration; ClinTox, Clinical Toxicity; FDA, Food and Drug Administration

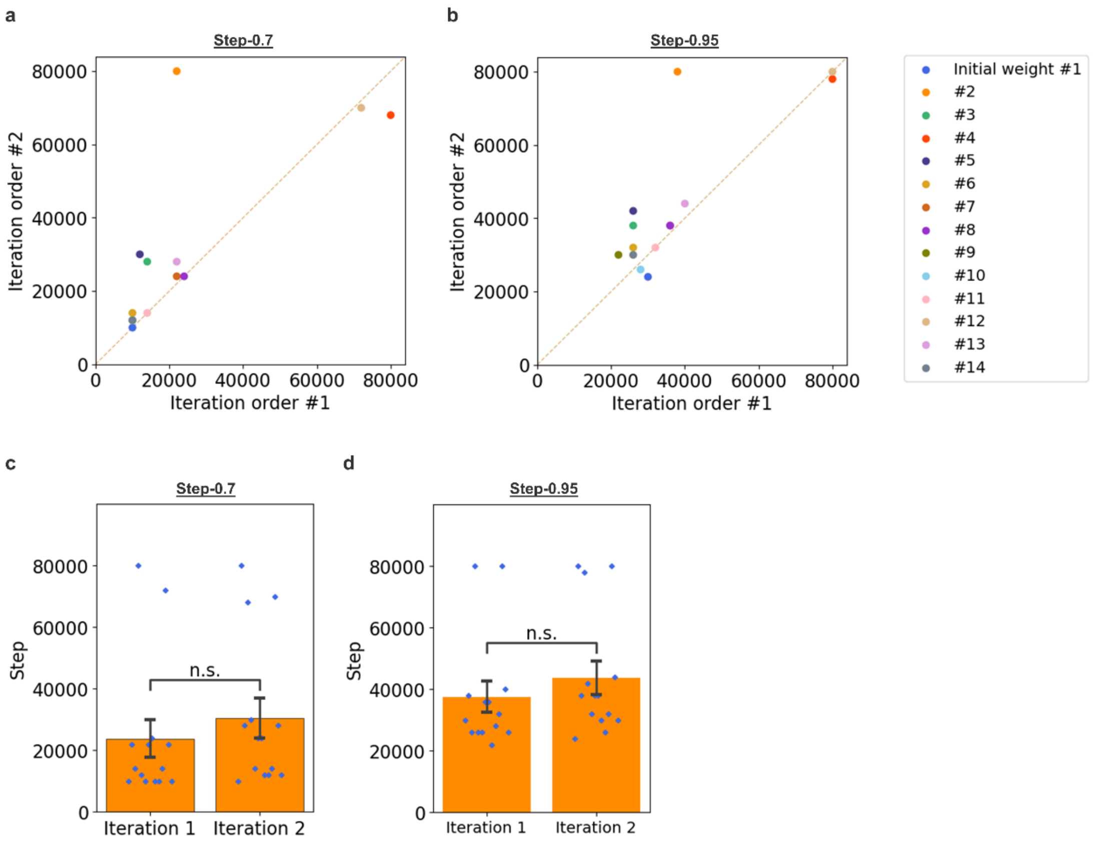

Supplementary Figure 3. Trainings with different initial weights and iteration orders

(a)(b) Comparison of *step-0.7/0.95* for each of 14 initial weights between 2 iteration orders. P values are calculated based on t-distribution with n - 2 degrees of freedom assuming the population correlation coefficient is 0. (c)(d) Average *step-0.7/0.95* for 14 initial weights of 2 iteration orders. Unbiased standard deviation and data distribution are shown as error bar length and blue plots, respectively. n.s. means $p > 0.05$ according to two-sided Welch's t-test. Source data are provided as a Source Data file.

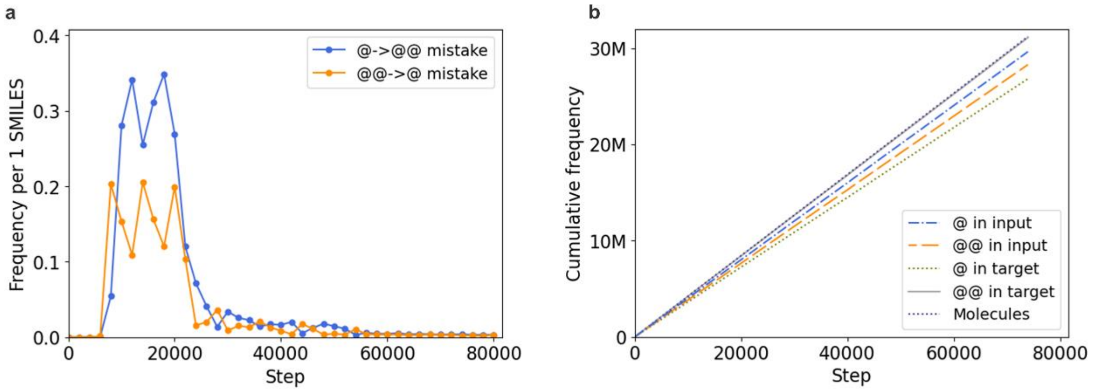

Supplementary Figure 4. Bias of "@" tokens and "@@" tokens

(a) Frequency of mistakes of "@" token for "@@" and "@@" token for "@" per one Simplified Molecular Input Line Entry System (SMILES) in the test set. Only mistakes in SMILES which was correctly predicted except chiral tokens are counted. (b) The accumulated number of molecules and "@" and "@@" tokens in each batch of the training set. Source data are provided as a Source Data file.

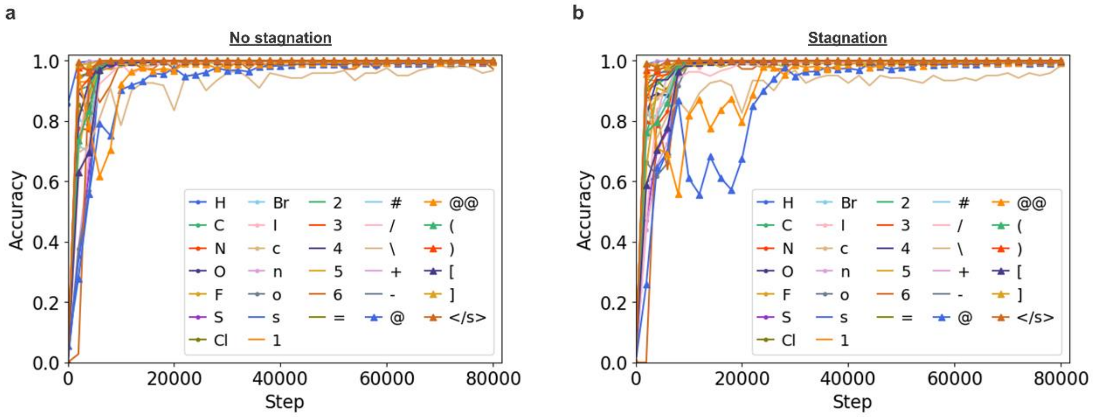

Supplementary Figure 5. Transition of character-wise accuracy with/without stagnation

(a) Transition of translation accuracy of each character when teacher-forcing was applied for a training without stagnation. (b) Transition of translation accuracy of each character when teacher-forcing was applied for a training with stagnation. Only characters with more than 10 appearances in the test set are shown. Source data are provided as a Source Data file.

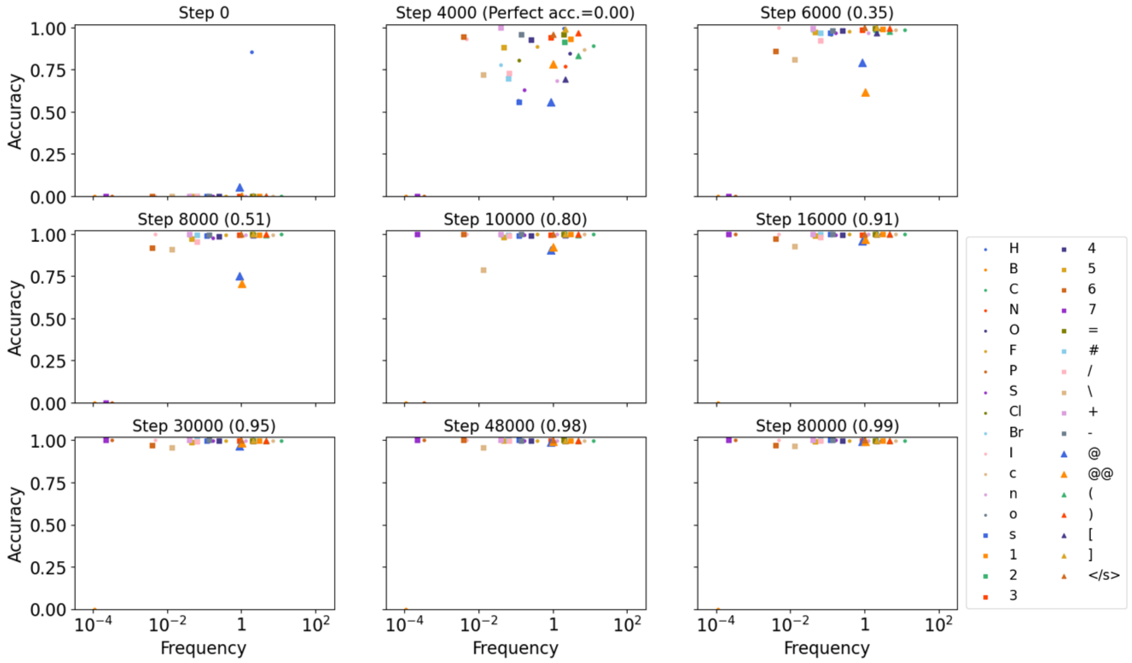

Supplementary Figure 6. Character-wise accuracy when stagnation did not occur

Translation accuracy of each character when teacher-forcing was applied for a training without stagnation. The horizontal axis shows the frequency in Simplified Molecular Input Line Entry System (SMILES) strings of the validation set, and the vertical axis shows the accuracy. Rare tokens which did not appear in the test set are not shown. Source data are provided as a Source Data file.

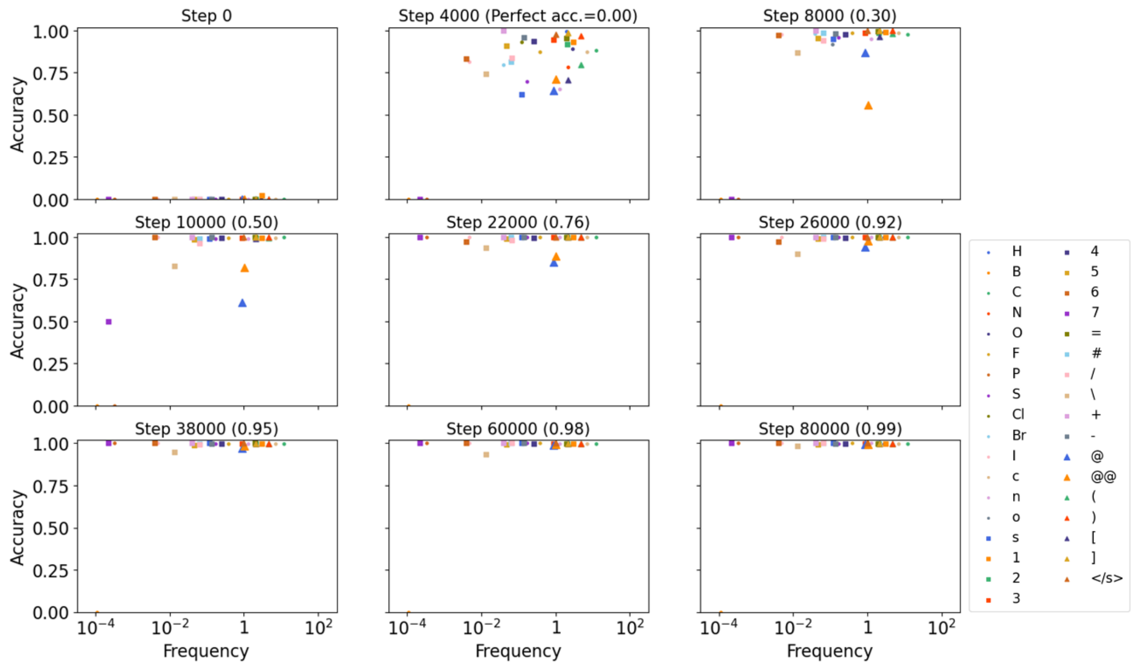

Supplementary Figure 7. Character-wise accuracy when stagnation occurred

Translation accuracy of each character when teacher-forcing was applied for a training with stagnation. The horizontal axis shows the frequency in Simplified Molecular Input Line Entry System (SMILES) strings of the validation set, and the vertical axis shows the accuracy. Rare tokens which did not appear in the test set are not shown. Source data are provided as a Source Data file.

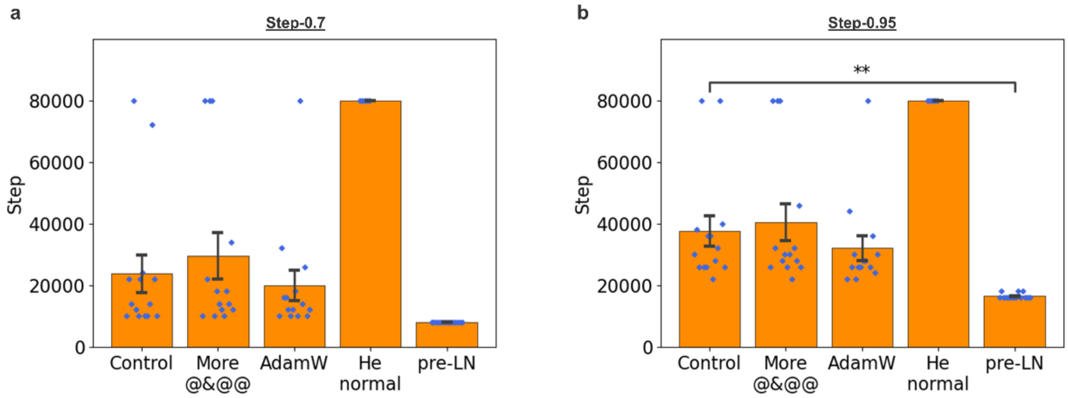

Supplementary Figure 8. Step-0.7/0.95 when 4 perturbation were applied

(a)(b) Average *step-0.7/0.95* when each of 4 perturbations was applied to 14 (or 5 for He normal) different initial weights. Unbiased standard deviation and data distribution are shown as error bar length and blue plots, respectively. ** means $p < 0.005$ according to two-sided Welch's t-test with Bonferroni correction. Source data are provided as a Source Data file.

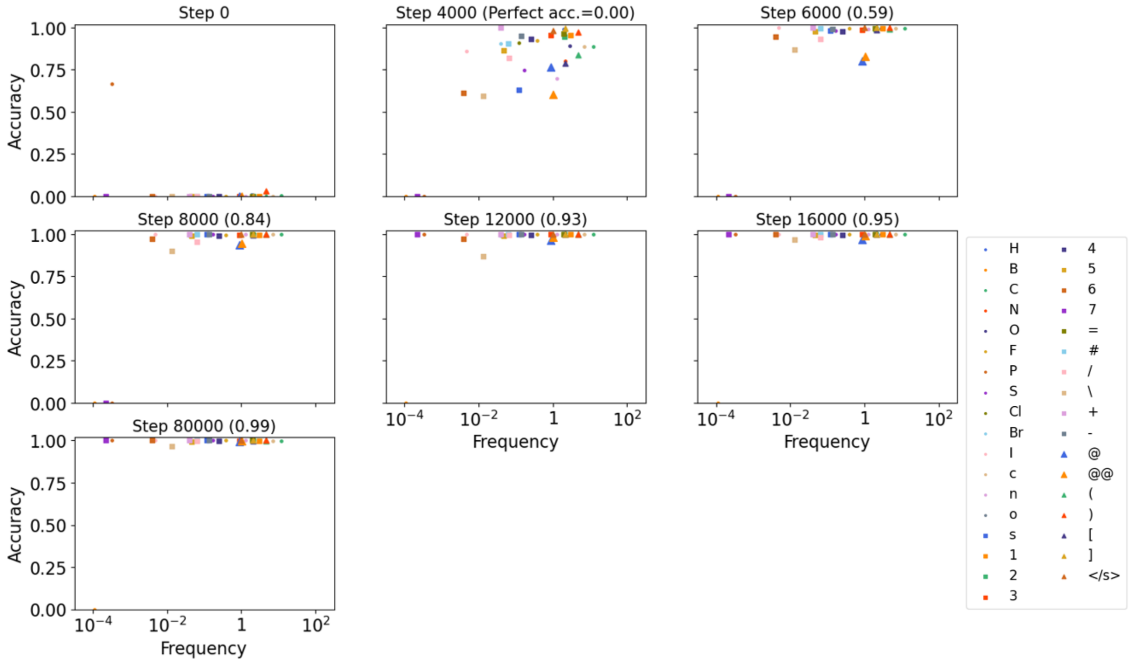

Supplementary Figure 9. Character-wise accuracy when pre-LN was introduced

Translation accuracy of each character when teacher-forcing was applied when the pre-Layer Normalization (pre-LN) structure was used. The horizontal axis shows the frequency in Simplified Molecular Input Line Entry System (SMILES) strings of the validation set, and the vertical axis shows the accuracy. Rare tokens which did not appear in the test set are not shown. Source data are provided as a Source Data file.

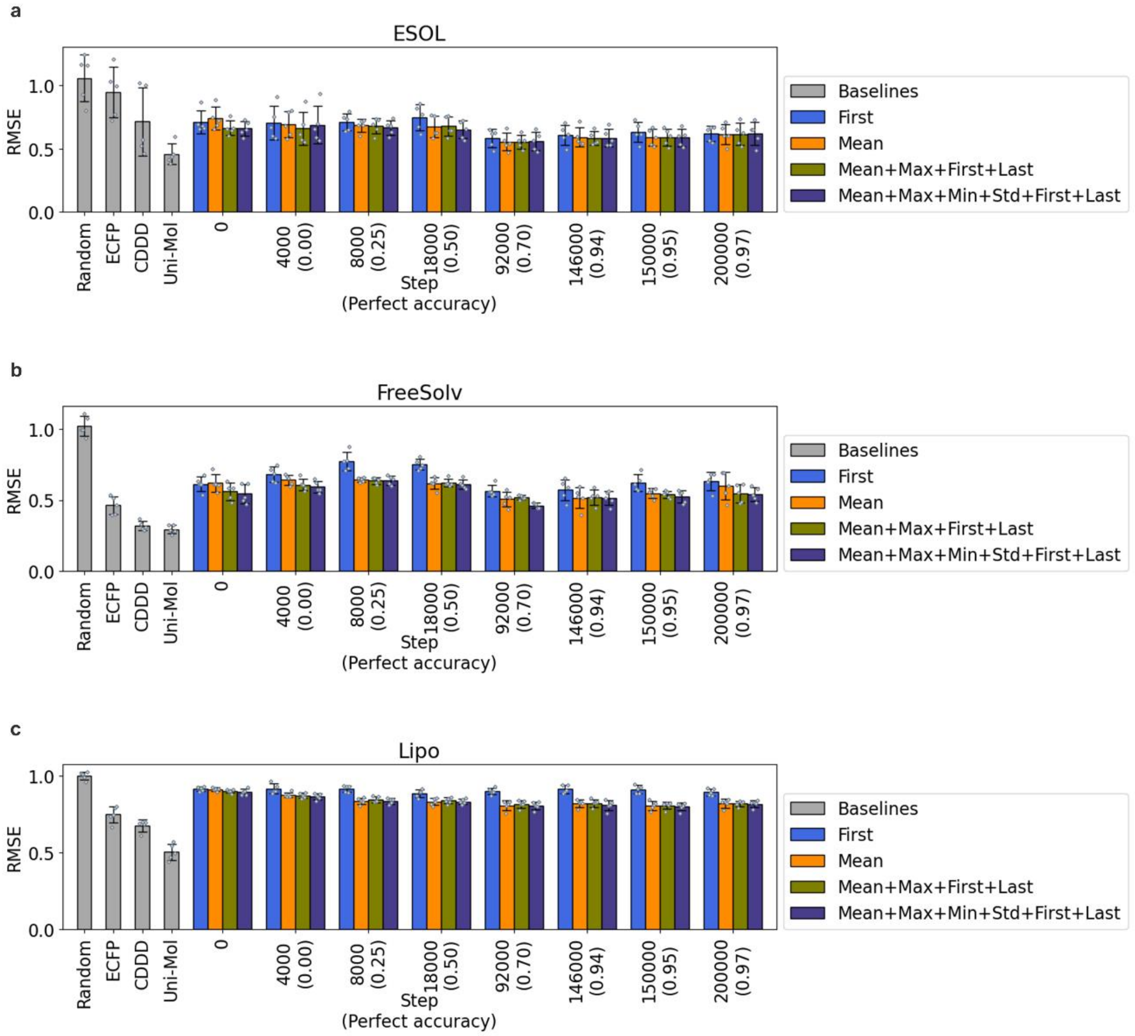

Supplementary Figure 10. Performance of descriptors from the model trained by InChI-to-SMILES translation on molecular property prediction (Regression task)

(a) RMSE score of prediction on ESOL dataset from descriptors of the model at different steps of training for 4 different ways of pooling. Blue, mean; yellow, latent representation of the first token; red, concatenation of the indicated 4 aggregation methods; navy, concatenation of the indicated 6 aggregation methods. (b) RMSE score of prediction on FreeSolv dataset from descriptors of the model at different steps of training for 4 different ways of pooling. (c) RMSE score of prediction on Lipophilicity dataset from descriptors of the model at different steps of training for 4 different ways of pooling. Mean, unbiased standard deviation and data distribution of experiments for 5 folds split by recommended method in DeepChem[31] are shown as bar height, error bar length and gray dots, respectively. The metrics were determined based on MoleculeNet[32]. Perfect accuracy at each step is written down on the horizontal axis. Source data are provided as a Source Data file. InChI, International Chemical Identifier; SMILES, Simplified Molecular Input Line Entry System; RMSE, Root Mean Squared Error; ESOL, Estimated Solubility; FreeSolv, Free Solvation Database;

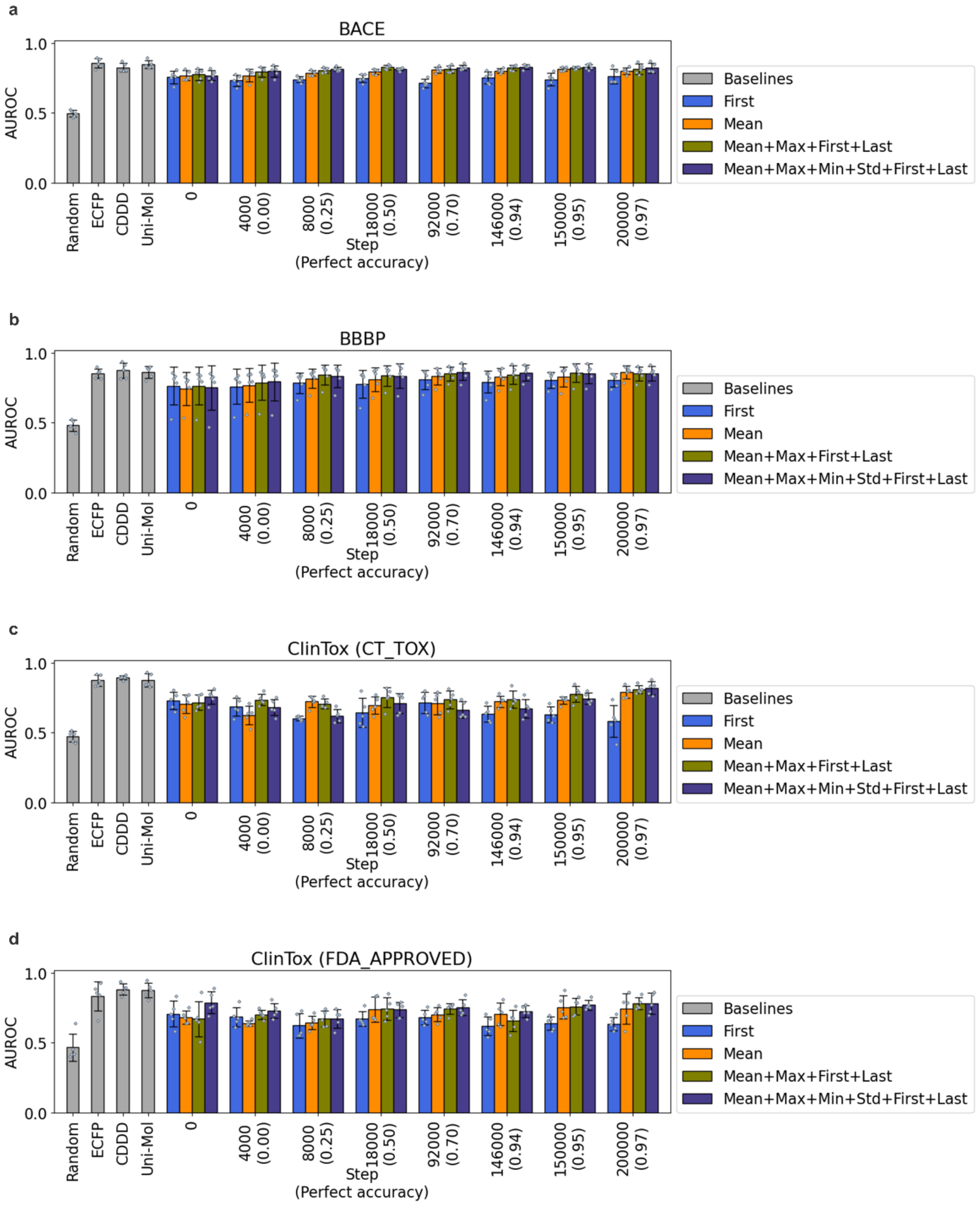

Supplementary Figure 11. Performance of descriptors from the model trained by InChI-to-SMILES translation on molecular property prediction (Classification task)

(a) AUROC score of prediction on BACE dataset from descriptors of the model at different steps of training, for 4 different ways of pooling. Blue, mean; yellow, latent representation of the first token; red, concatenation of the indicated 4 aggregation methods; navy, concatenation of the indicated 6 aggregation methods. (b) AUROC score of prediction on BBBP dataset from descriptors of the model at different steps of training, for 4 different ways of pooling. (c) AUROC score of prediction on ClinTox (failure of clinical trials for toxicity reasons) dataset from descriptors of the model at different steps of training for 4 different ways of pooling. (d) AUROC score of prediction on ClinTox (FDA approval) dataset from descriptors of the model at different steps of training for 4 different ways of pooling. Mean, unbiased standard deviation and data distribution of experiments for 5 folds split by recommended method in DeepChem[31] are shown

as bar height, error bar length and gray dots, respectively. The metrics were determined based on MoleculeNet[32]. Perfect accuracy at each step is written down on the horizontal axis. Source data are provided as a Source Data file. InChI, International Chemical Identifier; SMILES, Simplified Molecular Input Line Entry System; AUROC, Area Under Receiver Operating Characteristic; BACE, Inhibitors of human beta-secretase 1; BBBP, The blood-brain barrier penetration; ClinTox, Clinical Toxicity; FDA, Food and Drug Administration

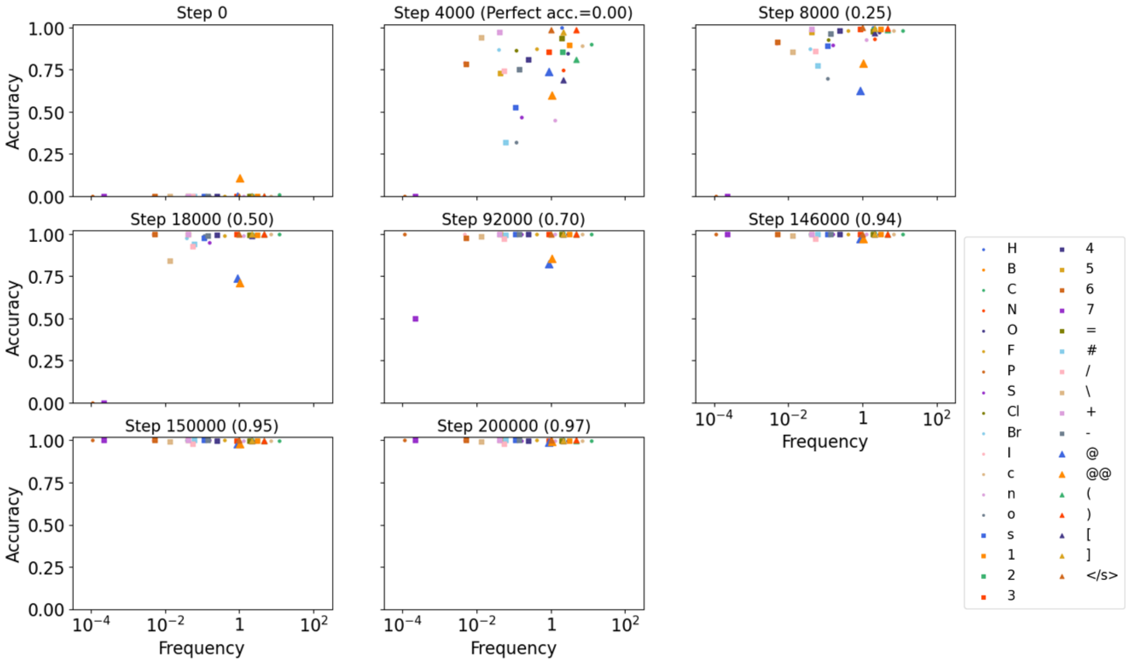

Supplementary Figure 12. Character-wise accuracy of the model trained with InChI-to-SMILES translation

Translation accuracy of each character when the model was trained to translate InChI into canonical SMILES, and teacher-forcing was applied. The horizontal axis shows the frequency in SMILES strings of the validation set, and the vertical axis shows the accuracy. Rare tokens which did not appear in the test set are not shown. Source data are provided as a Source Data file. InChI, International Chemical Identifier; SMILES, Simplified Molecular Input Line Entry System

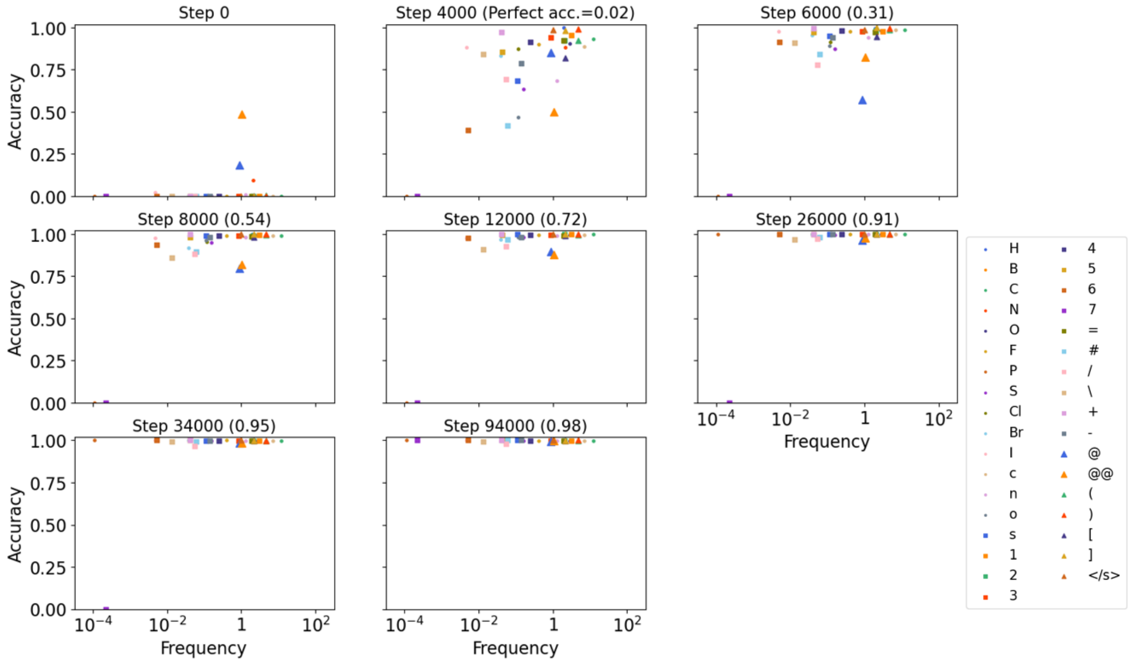

Supplementary Figure 13. Character-wise accuracy of the model with pre-LN structure trained with InChI-to-SMILES translation

Translation accuracy of each character when the model with pre-LN structure was trained to translate InChI into canonical SMILES, and teacher-forcing was applied. The horizontal axis shows the frequency in SMILES strings of the validation set, and the vertical axis shows the accuracy. Rare tokens which did not appear in the test set are not shown. Source data are provided as a Source Data file. InChI, International Chemical Identifier; SMILES, Simplified Molecular Input Line Entry System; pre-LN, Pre-Layer Normalization

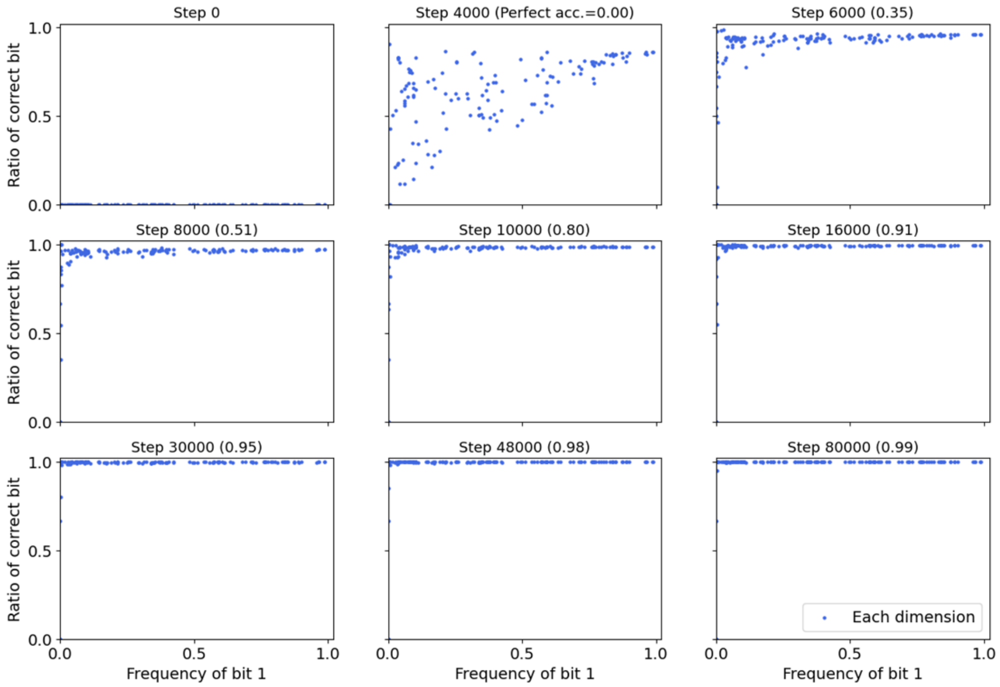

Supplementary Figure 14. Dimension-wise accuracy of MACCS keys

The ratio of molecules whose Simplified Molecular Input Line Entry System (SMILES) were validly decoded, and whose predicted/target molecules both have 1, to the number of molecules whose Molecular ACCess System (MACCS) keys have 1 in each dimension. The horizontal axis shows the frequency of bit 1, and the vertical axis shows the accuracy. Source data are provided as a Source Data file.

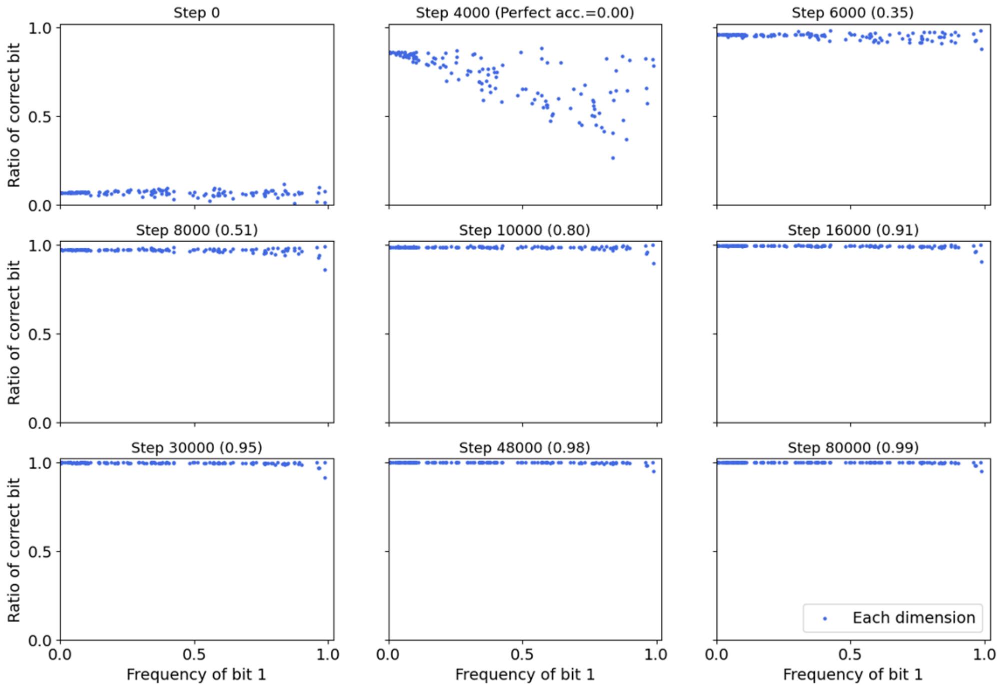

Supplementary Figure 15. Dimension-wise accuracy of MACCS keys

The ratio of molecules whose Simplified Molecular Input Line Entry System (SMILES) were validly decoded, and whose predicted/target molecules both have 0, to the number of molecules whose Molecular ACCess System (MACCS) keys have 0 in each dimension. The horizontal axis shows the frequency of bit 0, and the vertical axis shows the accuracy. Source data are provided as a Source Data file.

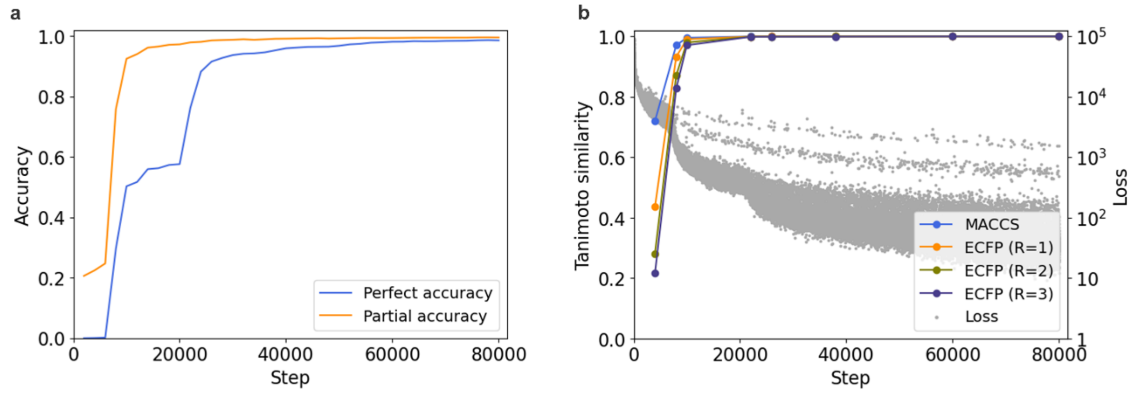

Supplementary Figure 16. Temporal change of Tanimoto similarity when stagnation occurred
(a) Temporal change of perfect/partial accuracy in the training case we used here. (b) Temporal change of Tanimoto similarity between fingerprints of predicted and target Simplified Molecular Input Line Entry System (SMILES) compared to loss function. Each gray dot indicates the loss of each batch. Source data are provided as a Source Data file.

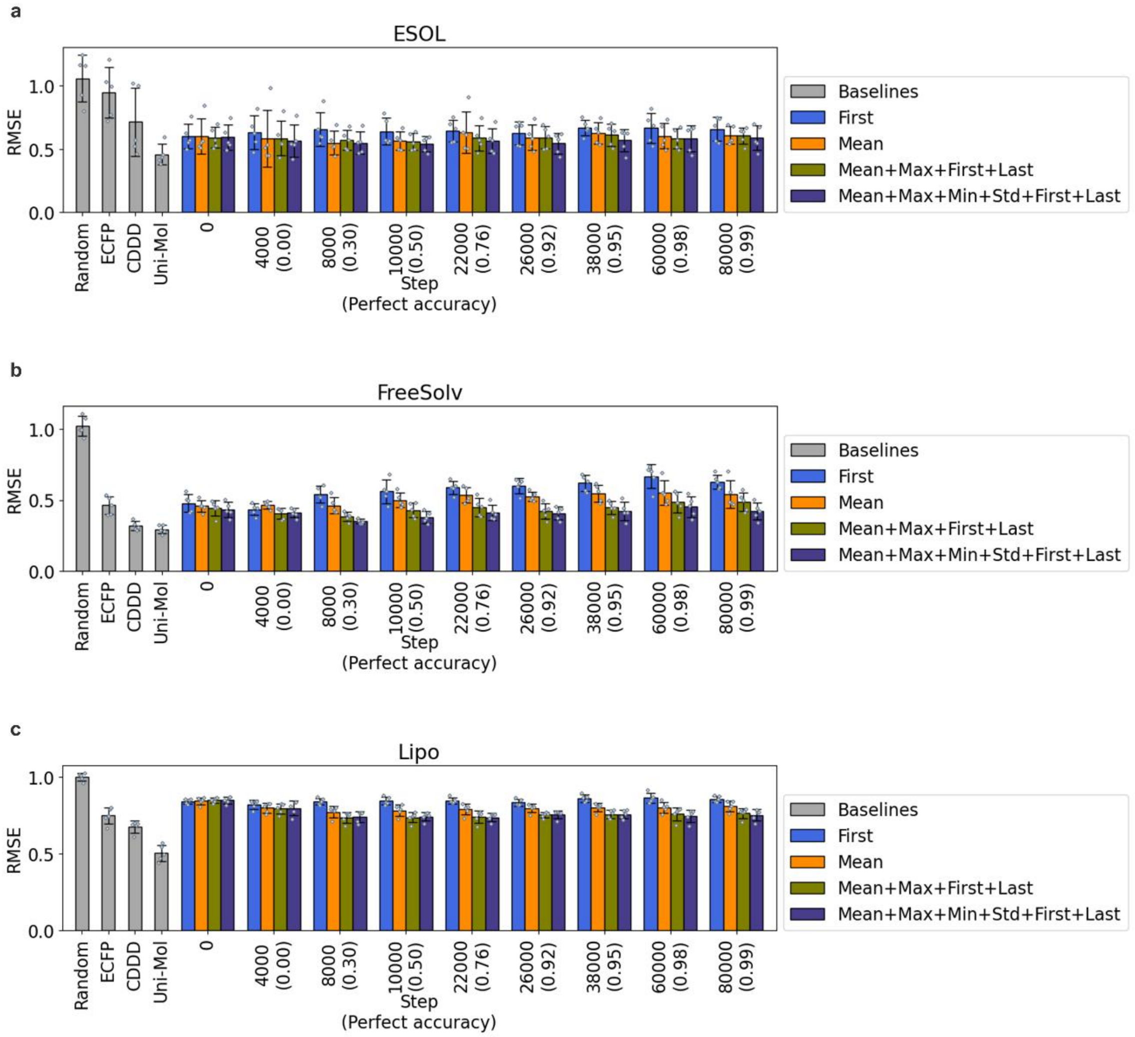

Supplementary Figure 17. Performance of descriptors on molecular property prediction (Regression task)

(a) RMSE score of prediction on ESOL dataset from descriptors of the model at different steps of training for 4 different ways of pooling. Blue, mean; yellow, latent representation of the first token; red, concatenation of the indicated 4 aggregation methods; navy, concatenation of the indicated 6 aggregation methods. (b) RMSE score of prediction on FreeSolv dataset from descriptors of the model at different steps of training for 4 different ways of pooling. (c) RMSE score of prediction on Lipophilicity dataset from descriptors of the model at different steps of training for 4 different ways of pooling. Mean, unbiased standard deviation and data distribution of experiments for 5 folds split by recommended method in DeepChem[31] are shown as bar height, error bar length and gray dots, respectively. The metrics were determined based on MoleculeNet[32]. Perfect accuracy at each step is written down on the horizontal axis. Source data are provided as a Source Data file. RMSE, Root Mean Squared Error; ESOL, Estimated Solubility; FreeSolv, Free Solvation Database;

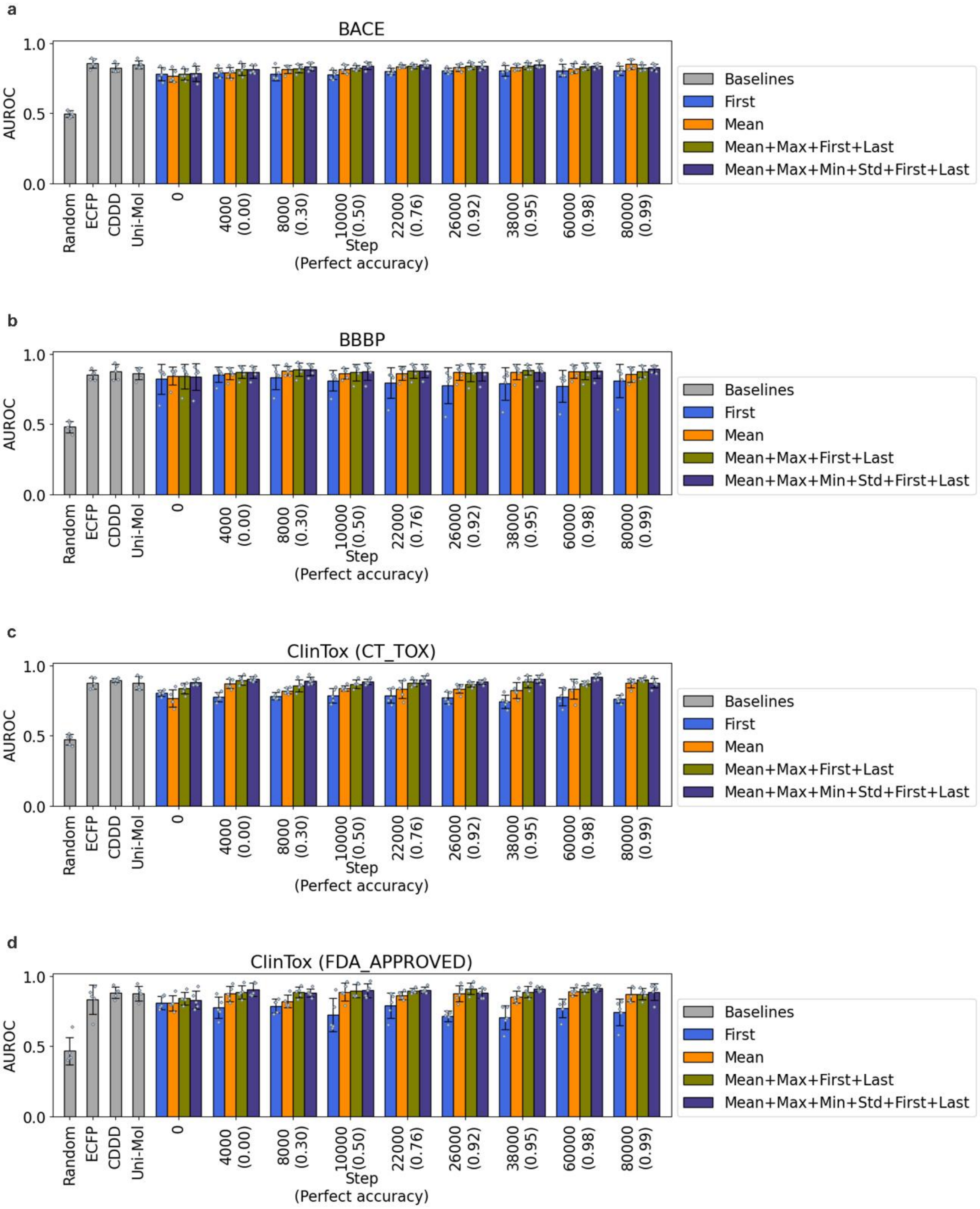

Supplementary Figure 18. Performance of descriptors on molecular property prediction (Classification task)

(a) AUROC score of prediction on BACE dataset from descriptors of the model at different steps of training, for 4 different ways of pooling. Blue, mean; yellow, latent representation of the first token; red, concatenation of the indicated 4 aggregation methods; navy, concatenation of the indicated 6 aggregation methods. (b) AUROC score of prediction on BBBP dataset from descriptors of the model at different steps of training, for 4 different ways of pooling. (c) AUROC score of prediction on ClinTox (failure of clinical trials for toxicity reasons) dataset from descriptors of the model at different steps of training for 4 different ways of pooling. (d) AUROC score of prediction on ClinTox (FDA approval) dataset from descriptors of the model at different steps of training for 4 different ways of pooling. Mean, unbiased standard deviation and data distribution of experiments for 5 folds split by recommended method in DeepChem[31] are shown

as bar height, error bar length and gray dots, respectively. The metrics were determined based on MoleculeNet[32]. Perfect accuracy at each step is written down on the horizontal axis. Source data are provided as a Source Data file. AUROC, Area Under Receiver Operating Characteristic; BACE, Inhibitors of human beta-secretase 1; BBBP, The blood-brain barrier penetration; ClinTox, Clinical Toxicity; FDA, Food and Drug Administration

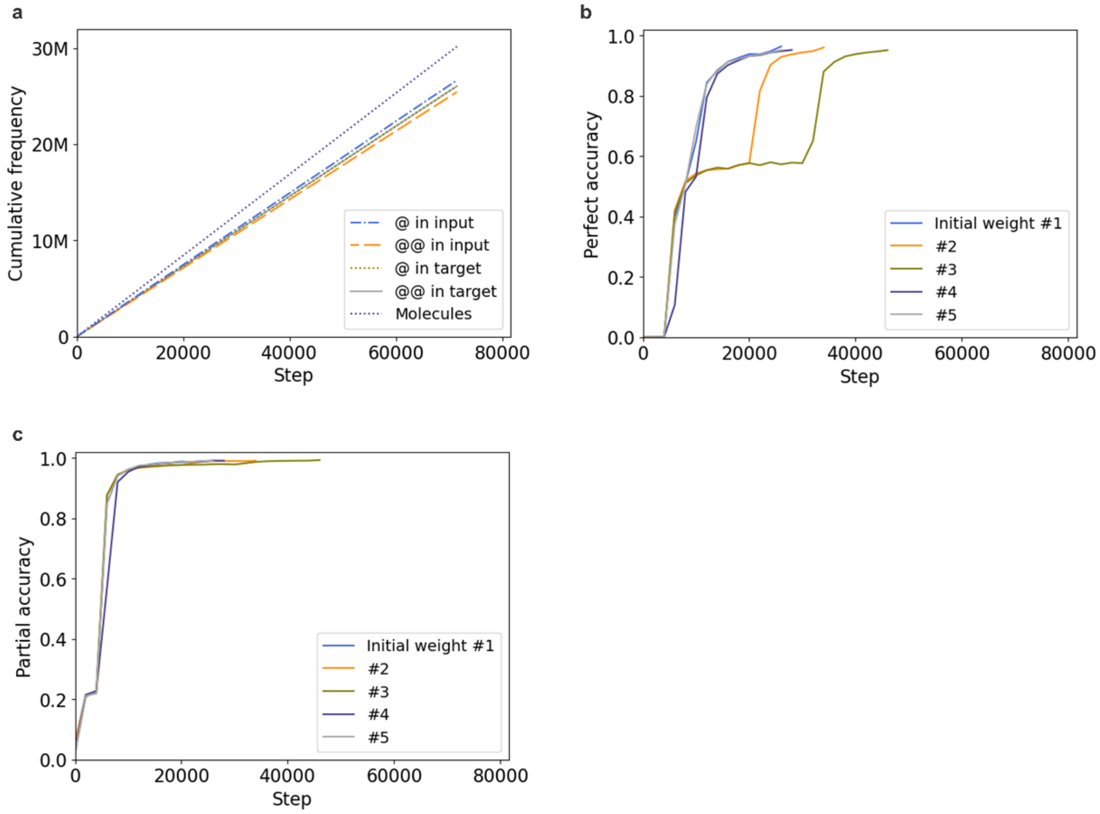

Supplementary Figure 19. Experiment with dataset containing equal number of chiral tokens

(a) The accumulated number of molecules and "@" and "@@" tokens in each batch of the training set which was downsampled so that the numbers of "@" and "@@" tokens are equal. (b) Temporal change of perfect accuracy of the model trained by the unbiased dataset started from 5 different initial weights. (c) Temporal change of perfect accuracy of the model trained by the unbiased dataset started from 5 different initial weights. Source data are provided as a Source Data file.

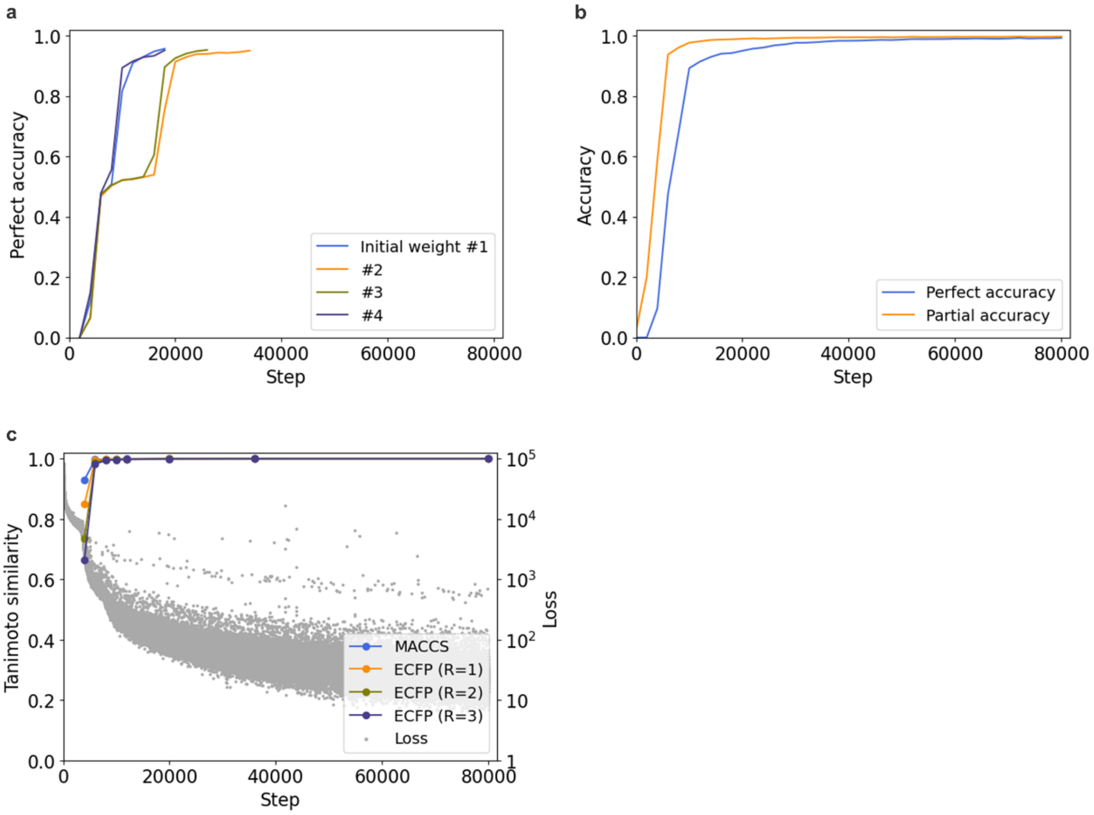

Supplementary Figure 20. Investigation of the effects of sampling strategy on the main results in this study

(a) Temporal change of perfect accuracy started from 4 different initial weights trained with randomly sampled molecules. (b) Perfect/partial accuracy of the training that was used for experiments. The model was trained with randomly sampled data. (c) Temporal change of Tanimoto similarity between fingerprints of predicted and target Simplified Molecular Input Line Entry System (SMILES) compared to loss function. Each gray dot indicates the loss of each batch. Source data are provided as a Source Data file.

Supplementary Tables

Supplementary Table 1. Vocabulary used to tokenize SMILES

Special tokens	<s> </s> <pad>
Normal tokens	H B C N O F P S Cl Br I c n o s 1 2 3 4 5 6 7 = / \ + - @ @@ ( ) [ ]

Supplementary Table 2. Vocabulary used to tokenize InChI

Special tokens	<s> </s> <pad>
Normal tokens	H B C N O F P S Cl Br I c i o n p s b l h t q m 1 2 3 4 5 6 7 8 9 = / \ + - . , : ? # % @ @@ ( ) [ ]

Supplementary References

1. Bagal, V., Aggarwal, R., Vinod, P. K. & Priyakumar, U. D. MolGPT: molecular generation using a transformer-decoder model. *J Chem Inf Model* **62**, 2064–2076 (2021).
2. Hong, Y.-B., Lee, K.-J., Heo, D. & Choi, H. Molecule Generation for Drug Discovery with New Transformer Architecture. Preprint at https://ssrn.com/abstract=4195528 (2022).
3. Rahimovich, D. R., Qaxramon O'g'li, A. S., O'g, S. R. A. & others. Application of transformer model architecture in the new drugs design. in *2021 International Conference on Information Science and Communications Technologies (ICISCT)* 1–3 (2021).
4. Shin, B., Park, S., Bak, J. & Ho, J. C. Controlled molecule generator for optimizing multiple chemical properties. in *Proceedings of the Conference on Health, Inference, and Learning* 146–153 (2021).
5. Kim, H., Na, J. & Lee, W. B. Generative chemical transformer: neural machine learning of molecular geometric structures from chemical language via attention. *J Chem Inf Model* **61**, 5804–5814 (2021).
6. Yang, Q. *et al.* Molecular transformer unifies reaction prediction and retrosynthesis across pharma chemical space. *Chemical communications* **55**, 12152–12155 (2019).
7. Karpov, P., Godin, G. & Tetko, I. V. A transformer model for retrosynthesis. in *International Conference on Artificial Neural Networks* 817–830 (2019).
8. Zheng, S., Rao, J., Zhang, Z., Xu, J. & Yang, Y. Predicting retrosynthetic reactions using self-corrected transformer neural networks. *J Chem Inf Model* **60**, 47–55 (2019).
9. Tetko, I. v, Karpov, P., van Deursen, R. & Godin, G. State-of-the-art augmented NLP transformer models for direct and single-step retrosynthesis. *Nat Commun* **11**, 5575 (2020).
10. Mao, K. *et al.* Molecular graph enhanced transformer for retrosynthesis prediction. *Neurocomputing* **457**, 193–202 (2021).
11. Maziarka, Ł. *et al.* Molecule attention transformer. Preprint at https://arxiv.org/abs/2002.08264 (2020).
12. Zhu, J. *et al.* Dual-view Molecule Pre-training. Preprint at http://arxiv.org/abs/2106.10234 (2021).
13. Shin, B., Park, S., Kang, K. & Ho, J. C. Self-Attention Based Molecule Representation for Predicting Drug-Target Interaction. in *Machine Learning for Healthcare Conference* vol. 106 230–248 (2019).
14. Chen, B., Barzilay, R. & Jaakkola, T. Path-augmented graph transformer network. Preprint at https://arxiv.org/abs/1905.12712 (2019).
15. Irwin, R., Dimitriadis, S., He, J. & Bjerrum, E. J. Chemformer: A pre-trained transformer for computational chemistry. *Mach Learn Sci Technol* **3**, 015022 (2022).
16. Gómez-Bombarelli, R. *et al.* Automatic chemical design using a data-driven continuous representation of molecules. *ACS Cent Sci* **4**, 268–276 (2018).
17. Kingma, D. P. & Welling, M. Auto-encoding variational bayes. Preprint at https://arxiv.org/abs/1312.6114 (2013).
18. Winter, R., Montanari, F., Noé, F. & Clevert, D.-A. Learning continuous and data-driven molecular descriptors by translating equivalent chemical representations. *Chem Sci* **10**, 1692–1701 (2019).
19. Vaswani, A. *et al.* Attention Is All You Need. in *Advances in Neural Information Processing Systems* (2017).
20. Devlin, J., Chang, M.-W., Lee, K., Kristina, T. & Language, A. I. BERT: Pre-training of Deep Bidirectional Transformers for Language. Preprint at https://arxiv.org/abs/1810.04805 (2018).
21. Lewis, M. *et al.* Bart: Denoising sequence-to-sequence pre-training for natural language generation, translation, and comprehension. Preprint at https://arxiv.org/abs/1910.13461 (2019).
22. Lan, Z. *et al.* ALBERT: A Lite BERT for Self-supervised Learning of Language Representations. Preprint at http://arxiv.org/abs/1909.11942 (2019).
23. Liu, Y. *et al.* Roberta: A robustly optimized bert pretraining approach. Preprint at https://arxiv.org/abs/1907.11692 (2019).

24. Honda, S., Shi, S. & Ueda, H. R. SMILES Transformer: Pre-trained Molecular Fingerprint for Low Data Drug Discovery. Preprint at http://arxiv.org/abs/1911.04738 (2019).
25. Fabian, B. *et al.* Molecular representation learning with language models and domain-relevant auxiliary tasks. Preprint at http://arxiv.org/abs/2011.13230 (2020).
26. Deng, D., Lei, Z., Hong, X., Zhang, R. & Zhou, F. Describe Molecules by a Heterogeneous Graph Neural Network with Transformer-like Attention for Supervised Property Predictions. *ACS Omega* **7**, 3713–3721 (2022).
27. Chen, J., Zheng, S., Song, Y., Rao, J. & Yang, Y. Learning attributed graph representations with communicative message passing transformer. Preprint at https://arxiv.org/abs/2107.08773 (2021).
28. Yoo, S. *et al.* Graph-Aware Transformer: Is Attention All Graphs Need? Preprint at http://arxiv.org/abs/2006.05213 (2020).
29. Pattanaik, L. *et al.* Message Passing Networks for Molecules with Tetrahedral Chirality. Preprint at http://arxiv.org/abs/2012.00094 (2020).
30. Adams, K., Pattanaik, L. & Coley, C. W. Learning 3D Representations of Molecular Chirality with Invariance to Bond Rotations. Preprint at http://arxiv.org/abs/2110.04383 (2021).
31. Ramsundar, B. *MOLECULAR MACHINE LEARNING WITH DEEPCHEM*. http://purl.stanford.edu/js264hd4826 (2018).
32. Wu, Z. *et al.* MoleculeNet: A benchmark for molecular machine learning. *Chem Sci* **9**, 513–530 (2018).
